# Single Room Maternity Care Versus Traditional Maternity Care: A Cross-Sectional Study Examining Differences in Mothers’ Perceptions of Readiness for Discharge and Satisfaction and Health Outcomes

**DOI:** 10.1177/08445621231165233

**Published:** 2023-03-21

**Authors:** Marc Hall, Arfan Afzal, Deborah E. White

**Affiliations:** 1 Faculty of Nursing, 2129 University of Calgary, Calgary, Canada; 2 2129 University of Calgary, Doha, Qatar

**Keywords:** pregnancy, maternity nursing, hospitals, mothers, delivery rooms

## Abstract

**Background:**

Single room maternity care (SRMC) includes all aspects of the birth process (labour, delivery, postpartum) in a single room with a consistent team of healthcare providers. Traditional maternity care (TMC) involves having mothers labouring and delivering their baby in one room and then transferring to a room on another unit, which also means a transition in providers. Although many hospitals have transitioned to SRMC, there has been limited evidence to support their development.

**Methods:**

This study was conducted in two large hospitals (one offering SRMC, the other TMC) in Western Canada. A cross-sectional between-subjects design was used to compare differences between SRMC and TMC. New mothers were asked to complete validated questionnaires. Health information was collected from administrative and health databases. The main outcomes included readiness for hospital discharge, mothers’ satisfaction, newborn length of stay, and mother length of stay. Several covariates were examined.

**Results:**

In total, 506 (292 SRMC; 214 TMC) mothers participated. Readiness for discharge and maternal satisfaction were significantly higher in SRMC. Although newborn and mother length of stay were significantly reduced in SRMC compared to TMC for univariate tests, mother length of stay was not significantly different when adjusting for other variables.

**Conclusions:**

There are positive health and psychosocial outcomes for mothers and newborns in the SRMC model of care compared to TMC. Since readiness for discharge and satisfaction are associated with positive maternal-infant interactions and transitions to community, SRMC could be the better approach. Further research should examine healthcare provider outcomes and implementation costs.

## Background and purpose

There has been a shift of viewing childbirth from a medicalized process to a normal family event in Western societies such as Canada, the United States, and parts of Europe (Waller-Wise, 2012). This approach goes beyond just caring about the physical needs of the mother and newborn, and incorporates the psychosocial aspects of care, as well as more fulsome integration of the family in care processes and decision-making. In Canada, it has been recommended to implement a single-room maternity care (SRMC) approach (Health Canada, 2000). As such, there has been a rise in the number of hospitals offering SRMC (Public Health Agency of Canada, 2012).

The change to family-centered approaches has roots from the 1970s feminist movement (Waller-Wise, 2012). This led to the conceptualization and implementation of SRMC starting in the 1980s (Phillips & Fenwick, 2000; Zwelling & Phillips, 2001). In SRMC, families remain in a single room for the duration of their stay. They labour, deliver, and recover all within the same room. They have the same team of healthcare providers (e.g., registered nurses, etc.) over the course of their stay (Gerrits et al., 2013; Phillips & Fenwick, 2000). In SRMC, nurses are cross-trained in all facets of care involved from when women are admitted to when they are discharged (Phillips & Fenwick, 2000). In traditional maternity care (TMC), families are transferred from an intrapartum unit for labour and delivery to a postpartum unit for recovery. The intrapartum and postpartum units have their own separate staff since nurses specialize in one type of maternity care (Stolte et al., 1994). One study of SRMC found that nurses described practices as relational and patient-centered where teaching, care, and support of families was apparent in every stage of the childbirth process (Ali et al., 2019). In contrast, in a study comparing SRMC and TMC units, providers described TMC as having a fragmentation of care and breakdown in communication and discharge planning (Hall et al., 2019). To note, other research demonstrates that quality of teaching influences mothers’ readiness for discharge (Şenol et al., 2017; Weiss & Lokken, 2009)

Although there is a shift towards SRMC units, there is limited evidence to support their development. A systematic review examining empirical studies of SRMC and TMC only yielded 13 studies, 3 of which were from the same hospital. The methodological quality of quantitative studies was weak, and few studies used inferential statistics to assess differences between maternity models (Ali et al., 2020).

In terms of satisfaction, a cross-sectional survey study found that mothers delivering in a new SRMC unit indicated they were satisfied with the care (Olson & Smith, 1992). A comparative study found that SRMC participants scored significantly higher (positively) compared to TMC participants in terms of adequacy of information and support received, privacy needs, physical environment, nursing care, teaching, infant feeding, and discharge planning (Janssen et al., 2000). In another study, scores for confidence in newborn care, postpartum nursing care, provision of choice, physical environment, respect for privacy, and labour and delivery nursing care were significantly higher in SRMC compared to TMC (Janssen et al., 2006). The former of these studies was descriptive and the two latter were conducted at the same hospital (Janssen et al., 2006; Janssen et al., 2000; Olson & Smith, 1992). None of these studies controlled for possible confounding health outcomes.

Studies examining clinical outcomes are few. One study examined clinical outcomes pre- and post-opening of a new SRMC unit (Harris et al., 2004). With the exception of lower rates of electronic fetal monitoring and intravenous therapy in SRMC, rates of intrapartum interventions and adverse outcomes were not significantly different. Length of stay was shorter in SRMC. Infant outcomes did not differ between groups, except for fewer 1-min Apgar scores <7 in SRMC. Another study reported fewer infants with hypoglycaemias in SRMC compared to TMC (Gerrits et al., 2013). Lastly, one study reported no change in perinatal mortality rate after SRMC was implemented (Williams & Mervis, 1990).

Several gaps remain about the impact of this approach. Many of the satisfaction studies were conducted at the same hospital in Western Canada. There have been limited studies on maternal and infant health outcomes (e.g., length of stay). An important element not yet explored is whether mothers feel more ready for discharge in SRMC compared to TMC. Studies indicate that new mothers who did not feel ready for discharge were less happy, asked more questions, were more likely to place their infants in prone sleeping position, and had greater coping difficulty within the first 3 weeks of discharge (Bernstein et al., 2002; Weiss & Lokken, 2009). Despite the importance of readiness for discharge, there have been no studies conducted to assess this in the SRMC literature.

The objectives of this study were to compare SRMC and TMC units in terms of 1) maternal perceptions of readiness for hospital discharge and satisfaction of hospital stay, and 2) maternal and infant health outcomes, while 3) examining possible confounding health outcomes.

## Methods and procedures

### Design and setting

We used a cross-sectional between-subjects design. The independent variable was type of maternity care (SRMC vs. TMC). Dependent variables included mothers’ readiness for hospital discharge and satisfaction, newborn length of stay, and mother length of stay. Several covariates were also examined.

The study was conducted in two large tertiary hospitals in a Western Canadian province. One hospital employed SRMC, the other TMC. For both hospitals, the nurse-patient ratio was 1:1 during delivery and 1:4 during postpartum. The SRMC hospital had 1 unit of 24 suites for labouring women to stay from admission to discharge. Rooms were private, spacious, and furnished to resemble a home-like environment. Partners were able to stay overnight. The average length of stay for mothers from admission to discharge at the SRMC hospital was 45.6 hours (based on administrative data from Feb 2016-Sept 2017). For the TMC hospital, women triaged in one room, laboured and delivered in another, and then were transferred to another unit for the duration of their postpartum stay. There were 12 labour and delivery beds in the labour and delivery unit. There were 20 postpartum beds in the postpartum unit, of which 15 were private (woman had a room to themselves) and 5 were semi-private (2 women shared a room). Rooms had shared showers and bathrooms. Partners could visit during hospital visitation hours.

The average length of stay for mothers from admission to discharge at the TMC hospital was 50.8 hours. For both hospitals, infants requiring neonatal care were transferred to a Neonatal Intensive Care Unit.

### Participants and recruitment

Recruitment occurred from March-October 2016. Mothers on both units were approached by a nurse within 12 hours of delivering their baby and asked if a research assistant could visit to describe the study. Those who consented were given an in-hospital questionnaire that took 5−10 minutes to complete. Participants were also asked to complete a post-discharge questionnaire that took 10 minutes. If participants chose the paper version, the questionnaire was provided in an envelope that included postage and return address and were instructed to complete and send the survey back 2 weeks post-discharge. If participants chose the electronic version, they were sent an e-mail 2 weeks post-discharge that included a link to the survey. Personal Healthcare Numbers were obtained from the unit clerk.

### Data collection

The in-hospital questionnaire included questions on age, education, ethnic/cultural background, language(s) spoken, whether they were born in Canada, marital status, and family income. Questions about breastfeeding, prenatal classes, using lactation consultants, and whether the baby stayed in the room were also included.

Readiness for discharge was measured using the 22-item Readiness for Hospital Discharge Scale which was part of the in-hospital questionnaire (Weiss & Lokken, 2009; Weiss & Piacentine, 2006). Questions were rated on an 11-point Likert-type scale, ranging from 0 =* not at all* to 11 =* totally*. The scale included 4 subscales of personal status, knowledge, coping ability, and expected support. Cronbach's α reliability estimates for the full scale were .92 and ranged from .81 to .86 for the subscales. Construct validity and predictive validity are supported for the scale.

Satisfaction was measured using the Care in Obstetrics: Measure for Testing Satisfaction Scale which was completed 2 weeks after discharge (Janssen et al., 2006). Forty questions were rated on varying 5-point Likert scales ranging from 1 to 5 (e.g., *strongly disagree* to *strongly agree*). The scale included 6 subscales of provision of choice, information, and support, physical environment, nursing care in labour, privacy, nursing care in postpartum, and confidence in newborn care. Cronbach's α reliability estimates for the full scale were .95 for the total scale, with subscales ranging from .82 to .91. Construct validity of the scale is supported by principal components factor analysis.

Administrative and health outcome data were collected from the provincial health authority. Data included the primary health outcomes of newborn and mother length of stay. Other health measures collected were used as covariates based on literature and expert knowledge. Antenatal risk assessment covariates included age >=35 years at delivery, hypertension >=140/90, other medical disorders, previous small for dates, diagnosis of small for dates, previous large for dates, diagnosis of large for dates, malpresentation, membranes ruptured before 37 weeks, pregnancy-induced hypertension, and substance use disorder. Intrapartum risk assessment covariates included meconium in labour, pregnancy-induced hypertension, fever, fetal heart rate abnormality, ruptured membranes >24 h, birth weight <2500 grams, baby admitted to NICU, gestational age <37 weeks, caesarean section delivery, excessive blood loss, stage-2 prolonged labour, and stage-3 prolonged labour. All data for covariates was coded as whether the variable was present or absent. We collected health outcome data for the sample of mothers as well as population data for comparison purposes. The sample health outcome data was collected from March-October 2016 (7 months) and the population health outcome data was collected from February 2016-September 2017 (20 months).

Ethical approval was granted through the Conjoint Health Research Ethics Board (REB14-1214). All Tri-Council Policy Statement guidelines were followed for obtaining consent, protecting confidentiality, and securely transferring and holding health data. Participants provided written informed consent.

### Analysis plan

Descriptive analysis was used to describe the demographic and clinical characteristics of the mothers. Categorical variables were expressed as frequencies and percentages and continuous variables were reported as median and range. Additionally, chi-square, Fisher's exact, or Mann-Whitney test, as appropriate, were used to differentiate the characteristics between the two types of maternity care.

Multiple linear regression model was completed to determine significant correlates of newborn and mother length of stay. Multivariate analysis of covariance (MANCOVA) was performed to determine if mother's readiness for discharge and satisfaction differed between the types of maternity care. We screened for demographic and clinical variables that could contribute to the response and included variables based on prior knowledge and literature. The percentage of missing values was minimal in all the models except for the satisfaction model, where over 40% of the responses were missing. However, Little's MCAR test (Little, 1988) indicated missing data was ‘missing completely at random’ (Chi-square: 436.28, df = 405, p = .14) and that complete case analysis would produce unbiased estimates. Therefore, we conducted complete case analyses. A family-wise correction was applied, where α was set at .01 for each regression model. No adjustment was made for the independent variables included in the analysis, as this would have substantially decreased the power of the tests. To address this and assess the stability of the regression coefficients, we used bootstrapping (Efron & Tibshirani, 1994) to examine our findings. In bootstrapping, observations from the original dataset are resampled to evaluate the reliability of a model. For our analysis, we resampled 1000 times to substantiate our results. The magnitude and significance status of the regression parameters from the bootstrapped model were consistent with the original model, indicating the validity of our findings.

Log-transformations of the outcomes (newborn and mother length of stay) were done for a linear relationship between the outcome and the independent variables. The assumptions of the linear regression models were tested using a normal P-P plot and a scatterplot of the residuals. The plots showed that the residuals are normally distributed and homoscedastic, suggesting that the linear regression models will generate accurate estimates. Prior to conducting the MANCOVA, a series of Pearson correlations were performed between all the items in each scale (readiness for discharge and satisfaction). A meaningful pattern of correlations was observed amongst the items, suggesting the appropriateness of a MANCOVA.

## Results

### Participants

In total, 516 mothers were recruited into the study (294 SRMC; 222 TMC). After initial data cleaning, 506 participants were included in analysis (292 SRMC; 214 TMC). Comparing health outcomes sample data to population data determined the sample was representative of the population characteristics of the mothers.

The SRMC hospital had significantly more mothers who spoke English as the first language (86% vs 58%), were Canadian citizens by birth (80% vs 53%), had higher family income (69% vs 50% with 85,000 CAD or more), and held the baby immediately after birth (82% vs 70%) compared to the TMC hospital (p < .01). As hypothesized, readiness for discharge and satisfaction were higher in SRMC, while the newborn length of stay and mother length of stay were lower (p < .001). A smaller proportion of mothers in SRMC had other medical disorders, fatal heart rate abnormality, and caesarean section compared to TMC (p < .001). However, more mothers experienced excessive blood loss and stage 3 prolonged labour in SRMC than TMC (p < .01) [Table 1].

**Table 1.** Characteristics of participants (socio-demographic, readiness for discharge, satisfaction, and clinical) from the two western Canadian hospitals, 2016 (n = 506).

Characteristics	Single Room Maternity	Traditional Maternity
	**n(%)**	**n(%)**
**Socio-demographic**	292 (57.7)	214 (42.3)
English as first language***	254 (86.4)	126 (57.5)
Born in Canada***	234 (79.9)	117 (53.4)
Family income (in CAD) ***		
Below 25,000	6 (2.1)	25 (11.6)
25,001−55,000	31 (10.8)	52 (24.2)
55,001−85,000	51 (17.8)	30 (14.0)
85,001 or more	199 (69.3)	108 (50.2)
Held baby immediately after delivery**	240 (81.9)	154 (70.0)
	**mean ± sd**	**mean ± sd**
**Readiness for discharge scale**		
Personal status***	60.7 **± **10.6	57.3 **± **10.0
Knowledge***	57.9 **± **8.7	55.1 **± **10.3
Coping ability***	25.5 **± **3.6	24.3 **± **3.8
Expected support*	36.2 **± **5.3	35.2 **± **5.4
**Satisfaction scale**		
Choice, information, and support***	32.0 **± **3.5	28.7 **± **5.2
Physical environment***	33.0 **± **2.3	26.9 **± **5.1
Nursing care in labour***	13.9 **± **1.7	12.1 **± **2.8
Privacy***	23.2 **± **2.3	20.7 **± **3.9
Nursing care in postpartum***	34.9 **± **5.5	29.2 **± **8.1
Confidence in newborn care***	43.9 **± **5.7	40.0 **± **8.3
**Clinical**		
	**median[range]**	**median[range]**
**Newborn length of stay** (in hours)***	35.3 [10.8−481.6]	45.8 [14.2−369.2]
**Maternal length of stay** (in hours)***	45.2 [2.8−617.8]	52.2 [16.8−138.6]
	**n (%)**	**n (%)**
**Antenatal risk assessment**		
Other medical disorders^a^***	4 (1.4)	29 (13.4)
**Intrapartum risk assessment**		
Fetal heart rate abnormality***	100 (36.0)	117 (54.4)
Caesarean section delivery***	80 (27.5)	97 (44.7)
Excessive blood loss^b^**	43 (14.8)	15 (6.9)
Stage-3 prolonged labour^c^**	20 (6.9)	4 (1.8)

*: p < .05, **:p < .01, ***:p < .001; a: other medical disorder includes epilepsy, severe asthma, lupus, Crohn's disease etc.; b: Excessive blood loss is defined as losing over 500 ml blood for vaginal delivery or over 1000 ml blood for caesarean section delivery; c: stage-3 prolonged labour is the duration of stage-3 labour over 15 min.

### Readiness for hospital discharge

A one-way multivariate analysis of covariance (MANCOVA) was conducted to test the hypothesis that maternity care type will differ in readiness for discharge after adjusting for other variables. The other variables included were marital status, family income, age, prenatal classes taken for this pregnancy, caesarean section, prolong stage-2 and stage-3 labour, lactation consult, length of stay of the mother and the baby, preterm birth, low birth weight and NICU admission of the baby. A statistically significant MANCOVA effect was obtained for maternity care (p < .001). The multivariate effect size was estimated as 0.12 implying 12% of the variance in the readiness for discharge was accounted for by the type of maternity care (specifically SRMC). Other variables that contributed to readiness were prenatal classes taken for this pregnancy (*p *< .001) and caesarean section (*p *< .001). The estimated effect size was largest for having a caesarean section delivery, which explained 25% of the variability in readiness. Taking prenatal classes for the current pregnancy also explained 16% of the variability of readiness. From MANCOVA, we found that SRMC was associated with a higher readiness score (*M *= 4.02, *SD *= 0.97) than TMC (*M *= 3.24, *SD *= 1.04). In accordance with Enders (2003), an independent two-sample T-test explored that readiness for discharge was significantly higher in SRMC compared to TMC (*p *< .001). To identify which items on the SRMC unit perform better, standardized discriminant function coefficients were extracted from the MANCOVA model. Coefficients with large absolute values correspond to variables with greater discriminating ability between the two types of maternity care [Table 2]. Specifically, mothers on the SRMC unit have more strength (0.30), less stress (0.49), better knowledge about caring for themselves (.78), their baby (0.32), and follow up care (.23), better ability to perform personal care (0.23), were expecting more support for household activities (.29), and were more emotionally ready to go home (0.35).

**Table 2.** Relative importance* of the major factors in differentiating single room maternity care and traditional maternity care in the readiness for discharge model (n = 461) and the participant satisfaction model (n = 265)

Readiness for Discharge Model (n = 461)						
Subscale	|Score|	Subscale	|Score|
**Personal status**		**Knowledge**
Physically ready to go home	0.19	Caring for yourself	0.78
Pain or discomfort	0.04	Caring for your baby	0.32
Strength	0.30	Problems to watch for	0.10
Energy	0.12	Who and when to call in a problem	0.06
Stress	0.49	Restrictions	0.17
Emotionally ready to go home	0.35	Follow up care	0.23
Physical ability to care for yourself	0.15	Services and information available to your community	0.09
Physical ability to care for your baby	0.16		
**Coping ability**		**Expected support**	
Handle the demands	0.05	Emotional support	0.10
Perform your personal care	0.23	Personal care	0.14
Perform baby care	0.05	Household activities	0.29
		Baby care	0.04
Participant Satisfaction Model (n = 265)			
Subscale	|Score|	Subscale	|Score|
**Choice, information and support**		**Physical environment**			
Caregiver helped viewing birth experience as a natural process	0.01	Room was specious and adequate	0.08
Asked about feeling/opinions in planning care	0.21	Able to find supplies needed	0.21
Information provided to make informed choice	0.03	Support person was comfortable	0.79
Supported decisions	0.12	Lighting was adequate	0.01
Assistance given to support person	0.19	Food was acceptable in quantity	0.14
Comfort measures given during labour	0.20	Food was acceptable in quality	0.08
Comfort measure given after the baby was born	0.07	Housekeeping staff respected privacy	0.23
**Nursing care in labour**		**Privacy**			
Time spent was sufficient for my physical need	0.21	Respect shown for privacy needs	0.07
Time spent was sufficient for my emotional need	0.27	Number of nursing looked after	0.001
Responded to needs in a timely manner	0.10	Numbers of doctors looked after	0.05
		Number of hospital staff came to the room during labour	0.02
		Number of hospital staff came to the room during postpartum	0.01
**Nursing care in postpartum**		**Confidence in newborn care**			
Time spent on physical need	0.07	Knowing when baby was hungry/full	0.002
Time spent on the emotional need	0.18	Knowing baby was getting enough milk	0.04
Time spent on feeding baby	0.24	Positioning baby to feed	0.07
Time spent on teaching how to take care of myself	0.06	Positioning baby to sleep	0.03
Time spent on teaching the support person about care	0.24	Knowing when baby was ill	0.07
Time spent on teaching how to take care of my baby	0.37	Knowing what to do when baby gags or chokes	0.06
Responded to needs in a timely manner	0.08	Giving baby a bath	0.32
Information received	0.09	Caring for baby's cord	0.07
		Coping with crying baby	0.08
		Finding sources of help in the community	0.10

* Relative importance is measured by the standardized discriminant function coefficients. An independent variable with a higher weight indicates the maternity cares were differentiated by this variable.

### Mother satisfaction

The potential predictors included in the satisfaction model were education, English as first language, age, held and breastfed baby immediately after delivery, length of stay, born in Canada, income, prenatal classes taken for current pregnancy, lactation consult, prolonged stage-2 and stage-3 labour of mother and maternity care type. The largest predictor of maternal satisfaction was the type of maternity care, with 59% of the variance accounted for by the type of care (*p *< .001). Taking prenatal classes for this pregnancy also contributed to satisfaction (*p *= .01) and explained 26% of the variability.

Mothers were significantly more satisfied with the care provided (Independent T-test, p < .001) in the SRMC hospital (M = 9.10, SD = 0.62) compared to the TMC hospital (M = 6.76, SD = 1.47). The standardized discriminant function coefficients in Table 2 suggests that the higher satisfaction of the SRMC mothers were mostly attributed to three *choice, information and support* items (asked about opinion in planning care, assistance given to support person, comfort given during labour), three *physical environment* items (able to find supplies, support person comfortable, housekeeping staff respected privacy), two *nursing care in labour* items (nurse spent sufficient time during labour for physical and emotional needs), four *postpartum care* items (nurse spent sufficient time during postpartum for emotional needs, feeding baby, teaching support person about care, teaching the mother how to take care of the baby) and one *confidence in newborn care* item (giving baby a bath).

### Newborn length of stay

The newborn's length of stay was significantly predicted by the SRMC, with a 13% decrease in the SRMC when compared to TMC [Table 3]. Also, 57% of the variability of log-transformed newborn's length of stay was explained by the 17 independent variables (adjusted R^2^=.57, p < .001). Not surprisingly, newborn's length of stay increased for preterm birth, NICU admission, caesarean delivery, and prolong stage-2 labour experienced by the mother.

**Table 3.** Factors predicting newborn's length of stay (n = 490) and mother's length of stay (n = 469) when comparing single room maternity care and traditional maternity care, western Canada, 2016

Variable	Coefficients (β)	SD(β)	t	p-value	exp(β)	CI of exp(β)
Newborn's LoS*						
*Intercept*	3.73	0.20	18.68			
*SRM care*	−0.14	0.03	−4.45	<.001	*0*.*87*	*[0.82, 0.92]*
*Preterm*	0.44	0.10	4.56	<.001	*1*.*55*	*[1.28, 1.88]*
*NICU admission*	1.01	0.09	11.67	<.001	*2*.*75*	*[2.32, 3.25]*
*Caesarean section*	0.51	0.04	14.57	<.001	*1*.*67*	*[1.55, 1.79]*
*Stage*-*2 prolonged labour^a^*	0.12	0.04	3.32	.001	*1*.*13*	*[1.05, 1.21]*
Mother's LoS**						
*Intercept*	3.55	0.07				
*SRM care*	-0.06	0.03	-2.12	.03	*0*.*94*	*[0.89, 1.00]*
*Newborn length of stay in days*	0.09	0.01	10.19	<.001	*1*.*09*	*[1.08, 1.12]*
*Caesarean section*	0.41	0.03	12.53	<.001	1.51	*[1.41, 1.60]*
*Stage*-*2 prolonged labour*	0.18	0.03	5.48	<.001	1.20	*[1.12, 1.27]*
*Fetal heart rate abnormality*	0.11	0.03	3.93	<.001	1.12	*[1.06, 1.19]*

*other covariates that are adjusted for: low birth weight, diagnosed small and large for dates, previous small for dates, stage-3 prolonged labour, malpresentation, meconium in labour, fever, fatal heart rate abnormality, delivery at 20–37 weeks, membranes ruptured before 37 weeks, ruptured membrane > 24 h; a:stage-2 prolonged labour is defined as over 30 min of labour at stage-2 for mothers with parity = 0 or over 60 min for mothers with parity >=1;

**other covariates that are adjusted for: Education, income, prenatal classes, preterm, diagnosed large for dates, stage-3 prolonged labour, malpresentation, meconium in labour, fever, age over 35 at delivery, other medical disorders, antenatal and intrapartum pregnancy-induced hypertension, excess blood loss.

### Mother length of stay

56% of the variability in the log-transformed mother's length of stay was explained by the independent variables (adjusted R^2 ^= 0.56, p < .001; Table 3). Having a caesarean section was the largest predictor, increasing mother's length of stay by 55% for mothers who had a caesarean section. Other significant predictors included newborn's length of stay, stage-2 prolonged labour, and fetal heart rate abnormality.

### Discussion

Postpartum is a transition period for the mother, father, and the newborn (Şenol et al., 2017). Having an environment that monitors newborn and maternal physical health and is supportive and fosters confidence in newborn care is key to empowering parents (World Health Organization, 2013). The purpose of this study was to compare mothers’ readiness for discharge and satisfaction between SRMC and TMC models and to explore health outcome differences between the models.

In this study, mothers in SRMC had significantly higher readiness for discharge scores (suggesting better knowledge, emotional readiness, and expected support). Janssen et al. (2000) reported significant differences when comparing nursing care, teaching and discharge planning of women on SRMC versus TMC units. In a study examining postpartum care, readiness for discharge was found to be higher for women who received information for their own and baby care and who would receive support for care when home (Şenol et al., 2017). Another study found that informational content explained 38% of the variance in discharge readiness for mothers (Weiss & Lokken, 2009). In this study, the SRMC model and prenatal classes explained 12% and 16% of the variance respectively for readiness. Previous work has shown that completion of a prenatal class significantly predicts the number of calls made to healthcare providers and the number of symptom days (total number of days with maternal or infant concerns), suggesting the information received in the classes increases readiness (Bernstein et al., 2013). Not surprisingly, caesarean section delivery explained 25% of the variance for readiness. Making the transition to home and community may be easier for mothers who feel more ready for discharge, since it has been found readiness helps mothers cope better post-discharge (Weiss & Lokken, 2009).

Maternal satisfaction is a frequently reported outcome measure for quality of care (Panth & Kafle, 2018). Similar to our findings, another study found higher satisfaction for mothers in SRMC in the areas of confidence in newborn care, delivery of nursing care, physical environment, and provision of choice and information (Janssen et al., 2000). In a systematic review of SRMC, mothers were found to have higher satisfaction with the SRMC model across studies (Ali et al., 2020). In this study, 59% of the variance in satisfaction was accounted for by the type of maternity care (SRMC), specifically the care to address both physical and emotional needs - needs that are often attributed to maternal competence and self efficacy (Bagherinia et al., 2018). Emotional and physical support needs have predicted maternal satisfaction during the postpartum period (Takács et al., 2015). In this study, taking prenatal classes for this pregnancy contributed to maternal satisfaction and explained 26% of its variability. Interestingly, other literature has found that attending prenatal classes was associated with negative experiences of birth (Smarandache et al., 2016). As Smarandache et al. (2016) indicates, these inconsistencies in study findings could be due to differences in class content, delivery, duration, or other variables. Understanding the specific influence of prenatal classes would provide additional opportunities to ensure that mother and baby progress well during this important transition period. Finally, increased maternal satisfaction of nursing care can lead to promoting mother-infant attachment and interactions (Ghadery-Sefat et al., 2016). Future research could examine whether attachment styles and interactions are impacted by model of care.

Factors identified in the literature that influence newborn length of stay include gestational age, low birth weight, and neonatal complications (Cheruiyot, 2013). As expected, length of stay of infants was extended by maternal and infant factors (preterm birth and NICU admission). Caesarean delivery and prolong stage-2 labour were also significant correlates of length of stay.

Perinatal complications because of maternal comorbidities (Van Otterloo et al., 2018), intrapartum factors, neonatal characteristics, and mode of delivery (Blumenfeld et al., 2015) are risk factors that have been identified with extended length of stay. These findings are consistent with our study; newborn length of stay, fetal heart rate abnormality, prolonged 2^nd^ stage of labour, and caesarian section delivery were all associated with increasing length of stay for mothers.

### Limitations

While we know the items that differentiated the maternal scores, we are limited in our understanding of both providers’ perceptions and the in-depth specificity of the care. It was more difficult to recruit at the TMC hospital, resulting in uneven sample sizes. This may be because of differences in unit culture, nurses’ interest in approaching participants, or research assistant approaches to consenting patients. Another limitation was the over 40% of missing data when comparing maternal satisfaction between the models. Participants may have lost interest or forgot to complete the post-discharge survey. While the data still had sufficient statistical power to identify differences, the missingness could reduce the sample representativeness. This data may not reflect the experiences in other locations. Despite these limitations, this study addresses important gaps in the literature, including the examination of readiness for discharge, calculations of effect size, multivariate statistical analysis in modelling predictors, and the inclusion of covariates, all of which have been very limited or are not discussed at all in the existing literature (Ali et al., 2020).

## Conclusion

During the postpartum period there are physical, emotional, and informational needs which require skilled teaching by providers and supportive care by providers and family to foster maternal competence and confidence. The study findings support the hypothesis that SRMC care contributes to mothers’ readiness for discharge and maternal satisfaction. Examination of structures and processes to enhance readiness and satisfaction are deserving of further attention. Mitigation strategies of factors that influence labour and delivery care complications also offer an opportunity to reduce both maternal and infant length of stay. Although this study suggests the SRMC model provides better outcomes for mothers and newborns, further research on healthcare provider outcomes and costs associated with implementing SRMC compared to TMC should be conducted to understand these care models more comprehensively.
